# Control of crosslinking for tailoring collagen-based scaffolds stability and mechanics

**DOI:** 10.1016/j.actbio.2015.07.034

**Published:** 2015-10-01

**Authors:** N. Davidenko, C.F. Schuster, D.V. Bax, N. Raynal, R.W. Farndale, S.M. Best, R.E. Cameron

**Affiliations:** a Department of Materials Science and Metallurgy, University of Cambridge, 27 Charles Babbage Road, Cambridge CB3 0FS, United Kingdom; b Department of Biochemistry, University of Cambridge, Downing Site, Cambridge CB2 1QW, United Kingdom

**Keywords:** Tissue engineering, Scaffolds, Collagen, Gelatin, Crosslinking

## Abstract

We provide evidence to show that the standard reactant concentrations used in tissue engineering to cross-link collagen-based scaffolds are up to 100 times higher than required for mechanical integrity in service, and stability against degradation in an aqueous environment. We demonstrate this with a detailed and systematic study by comparing scaffolds made from (a) collagen from two different suppliers, (b) gelatin (a partially denatured collagen) and (c) 50% collagen–50% gelatin mixtures. The materials were processed, using lyophilisation, to produce homogeneous, highly porous scaffolds with isotropic architectures and pore diameters ranging from 130 to 260 μm. Scaffolds were cross-linked using a carbodiimide treatment, to establish the effect of the variations in crosslinking conditions (down to very low concentrations) on the morphology, swelling, degradation and mechanical properties of the scaffolds. Carbodiimide concentration of 11.5 mg/ml was defined as the standard (100%) and was progressively diluted down to 0.1%. It was found that 10-fold reduction in the carbodiimide content led to the significant increase (almost 4-fold) in the amount of free amine groups (primarily on collagen lysine residues) without compromising mechanics and stability in water of all resultant scaffolds. The importance of this finding is that, by reducing cross-linking, the corresponding cell-reactive carboxylate anions (collagen glutamate or aspartate residues) that are essential for integrin-mediated binding remain intact. Indeed, a 10-fold reduction in carbodiimide crosslinking resulted in near native-like cell attachment to collagen scaffolds. We have demonstrated that controlling the degree of cross-linking, and hence retaining native scaffold chemistry, offers a major step forward in the biological performance of collagen- and gelatin-based tissue engineering scaffolds.

**Statement of Significance:**

This work developed collagen and gelatine-based scaffolds with structural, material and biological properties suitable for use in myocardial tissue regeneration. The novelty and significance of this research consist in elucidating the effect of the composition, origin of collagen and crosslinking concentration on the scaffold physical and cell-binding characteristics. We demonstrate that the standard carbodiimide concentrations used to crosslink collagenous scaffolds are up to 100 times higher than required for mechanical integrity in service, and stability against dissolution. The importance of this finding is that, by reducing crosslinking, the corresponding cell-reactive carboxylate anions (essential for integrin-mediated binding) remain intact and the native scaffold chemistry is retained. This offers a major step forward in the biological performance of tissue engineered scaffolds.

## Graphical abstract

**Figure:**
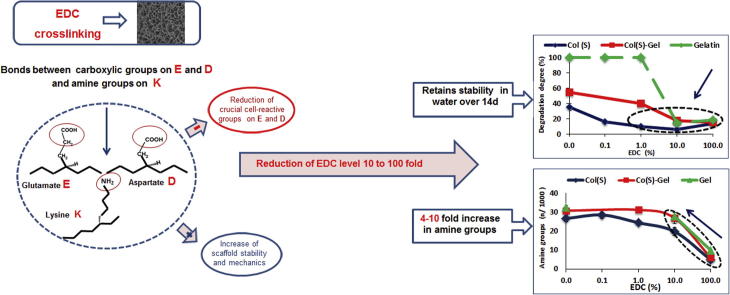

## 1 Introduction

The concept of tissue engineering (TE) has led to the utilisation of 3D scaffolds as cell-delivery vehicles [1–6]. Ideally, these biomaterials should mimic the function of the extracellular matrix (ECM) which not only provides mechanical support for cells, but also supplies signals that direct cell attachment, proliferation, differentiation and metabolism [7,8]. The main objective of this research is to tailor the material properties of collagen-, gelatin- and collagen–gelatin-based scaffolds by changing their composition and crosslink status. Collagen (Col) and gelatin (Gel) were selected as the main components for 3D scaffolds since, in many senses; they are ideal materials for soft tissue engineering constructs. There is a considerable amount of literature in the field already [1–3,6,9,10] and these works are described are normally based on a standardised route for cross-linking [6,10–12]. However, while this cross-linking step stabilises the mechanical- and degradation properties of the materials in service, the major drawback is the detrimental effects this may have on the biological activity of the scaffold [9,11,12].

Collagen is the most abundant protein in the ECM of many hard and soft tissues in the human body [13,14]. As examples collagen accounts for 90% of bone matrix protein content [15] and comprises up to 80% of myocardial ECM [16,17]. Especially important is fibrillar Type I collagen which provides structural support to resident cells in both soft and hard tissues. Collagen possesses the repetitive array of receptor-recognition motifs, essential for cell interaction, where the most important is GFOGER (where G is glycine, F is phenylalanine, O is hydroxyproline, E is glutamate and R is arginine). This high affinity triple-helical motif interacts with cells via specific β_1_-binding integrins, especially α_1_β_1_, α_2_β_1_, α_10_β_1_, α_11_β_1_ [18–22].

Gelatin, thermally denatured collagen, possesses more disorganised structure which should affect material and mechanical stability of the resultant scaffolds. Denaturation alters the macromolecular order of collagen, but chemical composition is closely maintained [22–24]. The destruction of the triple-helical system and the formation of random-coiled domains in gelatin also change the availability of cell-binding sites. Cell binding to the conformationally sensitive GFOGER motif is lost and instead the main receptor-recognition motif presented to cells is RGD (where D is aspartate) which signals to other integrins (mainly α_5_β_1_ and α_v_β_3_) [19,25,26]. As such the addition of gelatin to collagen can tailor the material stability, swelling and mechanical properties of scaffolds and could be a design strategy for changing biochemical activity. Further means of controlling these properties could be through the variation of the crosslinking status of these protein matrices.

Native collagen possesses multiple inter- and intramolecular cross-links, so providing structural strength and durability to their matrices in different biological environments. Extracting and purifying methods applied for processing naturally derived collagen into useful forms markedly reduce its cross-linking density with the consequent impact on the relevant properties and biological response. To improve structural stability and mechanics of collagenous materials different chemical (aldehydes, isocyanates, carbodiimides, etc.) [9,11,27–31] and physical (ultraviolet irradiation, dehydrothermal treatments, etc.) [32–36] methods could be used, none of which is completely satisfactory. In this works the carbodiimide treatment, EDC (1-ethyl-3-(3-dimethylaminopropyl-carbodiimide hydrochloride) in the presence of NHS (N-hydroxy-succinimide), at different concentrations was selected for scaffold cross-linking (XL). This method constitutes one of the most successful chemical crosslinking treatments for collagen-based biomaterials aimed at TE applications as it is highly efficient, nontoxic (EDC/NHS do not take part in linkage), and the resultant by-products can be easily removed by washing [9–11,31]. But, as EDC uses free primary amino groups (on lysine residues) and cell-reactive carboxylate anions (on glutamate or aspartate residues) for cross-linking [9,11,31] this may diminish the availability of essential cell binding motifs on collagen-type materials.

One of the goals of this work was the evaluation of the effect of reducing the EDC concentration down to very low levels (up to 1000 times dilution with respect to standard condition: EDC concentration of 11.5 mg/ml with molar ratios EDC/NHS/COO^−^(Col) = 5/2/1, defined as 100%) on scaffolds mechanics, dissolution behaviour and cell reactivity to obtain matrices with a variety of material and biological properties. The biological performance of collagenous materials before and after crosslinking with different EDC concentrations was also assessed in this work by studying cell adhesion on thin films. These 2D systems were chosen as a model for 3D scaffold struts. Human fibrosarcoma (HT1080) and human platelets were selected for this biological study.

The other important issue addressed in this study was the possible influence of the nature of the employed collagen on the relevant characteristics of the resultant scaffolds (morphology, dissolution resistance, mechanics, etc.). For this reason, Col-based and mixed scaffolds were produced using two types of microfibrillar Col I obtained from different providers: Sigma (S) and Devro (D) (Sigma–Aldrich Co. Ltd. UK and DevroMedical, respectively). Both were of bovine origin but derived from different tissues: Sigma Col was obtained from Achilles tendon while Devro sample was extracted from the epidermis layer in skin. This suggests that different processing/purifying methods were used for their production which may unequally affect essential characteristics of their macromolecular triple-helical structure (fibril lengths, width, entanglement, etc.) with the consequent implication on the final properties of the scaffolds obtained from these collagens. The effect of collagen origin on the behaviour of scaffolds produced from these collagen samples after EDC treatment was evaluated on their morphology, dissolution properties and resistance under compression.

## 2 Materials and methods

### 2.1 Materials

Two type I microfibrillar collagens were used in this work. One was derived from bovine dermal tissue (purchased from DevroMedical) and the other from bovine Achilles tendon (obtained from Sigma–Aldrich Co. Ltd., UK). Gelatin (type B from bovine skin, Gel), acetic acid (2 M), 1-ethyl-3-(3-dimethylaminopropyl) carbodiimidehydrochloride (EDC), N-hydroxy-succinimide (NHS) were purchased from Sigma Aldrich (UK). Other reagents were all analytical grade, commercially available and used as received.

### 2.2 Scaffold preparation

Protein scaffolds (Col, Gel and Col/Gel = 50/50%-wt.) were obtained by freeze-drying technique. Briefly, collagen was swollen in 0.05 M acetic acid at 4 °C overnight to produce a 1% (w/v) protein suspension. The resulting suspension was homogenised on ice for 30 min at 13,500 rpm using an Ultra-Turrax VD125 (VWR International Ltd., UK). Air bubbles were removed from the suspension by centrifuging at 2500 rpm for 5 min (Hermle Z300, Labortechnik, Germany). Gelatin solution, 1% (w/v), was prepared by dissolving gelatin in 0.05 M acetic acid at 37–45 °C with stirring for 1 h (h). The solution was then cooled to room temperature with stirring. The mixed collagen–gelatin 1:1 suspension was prepared by mixing a gelatin solution with a collagen slurry (both 1% w/v), homogenising for 15 min and centrifuging at the same conditions as Col suspensions. All suspensions were then poured into silicone trays (Lakeland, UK), to ensure ease of removal, and freeze-dried in a VirTis adVantage bench-top freeze-drier (Biopharma Process Systems, UK) using a cycle adapted from our previous work [10]. A constant cooling rate of 0.9 °C/min to a final freezing temperature of −26 °C was used. The temperature was then held constant at −26 °C for 90 min. The ice phase was sublimed for 20 h under a vacuum of less than 100 mTorr.

### 2.3 Scaffold crosslinking

Protein scaffolds (Col, Gel and Col/Gel = 50/50) were chemically cross-linked using a water-soluble carbodiimide (EDC) in presence of NHS at different concentrations. EDC concentration of 11.5 mg/ml of ethanol/water (75% v/v) and with molar ratios EDC/NHS/COO^−^(Col) = 5/2/1 was defined as the standard condition (100%). These cross-linking parameters (corresponding to 100%) were selected on the basis of our previous results and also on the reports from literature [6,10,11,37] recommended this cross-linking condition as one of the best to achieve the highest degrees of mechanical and material stabilisation for collagen-type matrices. To obtain scaffolds with different cross-linked states the EDC concentration was serially diluted in 10-fold steps from 100% to 0.1%. After reaction in the corresponding EDC/NHS solution for 2 h at room temperature, the scaffolds were washed thoroughly in deionised water (15 min × 5) and were subsequently refrozen and re-lyophilized using the previous freeze-drying cycle.

### 2.4 Morphology and dimensional properties

#### 2.4.1 Morphology

Scanning electron microscopy (SEM) (JEOL 5800) was used to analyse the internal architecture of scaffolds with different compositions and XL status. Longitudinal and transverse cross-sections were cut from different part of scaffold sheet, mounted on stubs and sputter coated with a layer of platinum for observation at 10 keV at various levels of magnification. Images were used to compare the characteristics of scaffold porous structures (homogeneity, interconnectivity, etc.) and to determine their pore sizes by the manual measurement of different pore zones (at least 30 pores randomly chosen from different microphotographs of each sample).

#### 2.4.2 Dimensional properties

Possible influences of composition, Col nature (S vs D) and EDC treatment at standard concentration were assessed on the following dimensional properties of scaffold: Relative density (**ρ^∗^**/**ρ_s_**), calculated from the measured dry density of the scaffold post freeze-drying (**ρ^∗^** = **m**/**V**; where **m** is the mass and **V** is the volume of the 3D matrix) and the known dry density of solid collagen (gelatine) (**ρ_s_**), assumed to be 1.3 g ml^−1^) [28]. Porosity defined as the percentage of void space in a 3D sponge and determined from relative density values [38]:

$$\text{Porosity}\;(\%)=(1-\rho^{*}/\rho_{\text{s}})\times 100$$

### 2.5 Characterisation of collagen fibre structure

#### 2.5.1 Rheology

Viscosity measurements of 1% (w/v) protein slurries in 0.05 M acetic acid were carried out by ARES controlled strain Rheometer (TA Instruments, formerly Rheometrics Scientific Inc.) The tests were performed using either a parallel plate geometry (plate diameter 50 mm) or a couette geometry (cup diameter = 34 mm, bob diameter = 32 mm, bob length = 34 mm). The temperature was held at 20 °C (controlled by a water jacket).

#### 2.5.2 Confocal studies of peptide attachment to collagen fibres

In order to assess the architecture of collagen fibres slurries of 10 μg/ml of collagen (D and S) in 0.01 M acetic acid were prepared. Polymeric type I collagen from bovine tendon (Ethicon Inc., Somerville NJ, USA), Ethicon, and soluble monomeric Col from calf skin (Devro pty), were employed in the same concentrations as positive and negative controls, respectively. Triple-helical GFOGER peptide, used for attachment, was produced and fluorescein-labelled (FL) in the Biochemistry Department of Cambridge University (Farndale laboratory) as described in [39,40]. GFOGER-FL has an excitation wavelength of 492 nm and emission of 517 nm.

For observation by confocal microscopy, 100 μl of each collagen suspension were pipetted individually to wells of an 8-well chambered glass cover slip (Thermo Scientific – Lab Tech). After 2 h, the unbound solution was removed and 200 μl of block solution (50 mg/ml bovine albumin serum, BSA, in tri-buffered saline solution, TBS) was added to each well. After 1 h the block was removed and the wells were washed with 200 μl of 0.1% (v/v) Tween in TBS (×3). 100 μl of GFOGER-FL (10 μg/ml of 0.1% Tween in TBS) were added to the wells for attachment. After 1 h the solutions were removed and the wells rinsed with 0.1% (v/v) Tween in TBS (×3). 200 μl TBS were added to each well before observation in FTIC mode using an Olympus FV300 laser-scanning confocal microscope system, and IX81 inverted frame. Results were analysed using Olympus Fluoview v5.0 software.

### 2.6 Amine group content and degree of cross-linking

The content of free primary amine groups present in the initial non-crosslinked samples and those crosslinked with different EDC concentrations was determined using a 2,4,6-trinitro-benzene-sulfonic acid (TNBS) assay with a protocol similar to those reported by Sashidar et al. [41] and Ofner et al. [42]. To each sample (2–4 mg), 0.5 ml of a 4% (w/v) NaHCO_3_ solution and 0.5 ml of a freshly prepared solution of 0.05% (w/v) TNBS were added. After 2 h at 40 °C, 1.5 ml of 6 M HCl was added and the samples were hydrolysed at 60 °C for 90 min. The reaction mixture was diluted with distilled water (2.5 ml), cooled to room temperature, and A_320_ was measured using a Fluostar Optima spectrophotometer. Controls (blank samples) were prepared using the same procedure, except that HCl was added, prior to introducing the TNBS solution, to prohibit any reaction of TNBS with the amine groups. The blank sample value was subtracted from each sample absorbance. The absorbance was correlated to the concentration of free amine groups using a calibration curve obtained with glycine in an aqueous NaHCO_3_ solution. Three replicates were used for each determination and the number of amino groups per 1000 residues calculated. The non-XL sample was assumed to contain 100% of the available free amine groups and this value was used to calculate the percentage of remaining free amine groups after the crosslinking treatment. The degree of crosslinking was evaluated as the difference between the chemically-determined number of free amines groups before and after crosslinking, relative to the initial free amine content. In determining the degree of crosslinking, it has been assumed that each lost amine group participates in one crosslink.

### 2.7 Dissolution in distilled water

This study was carried out in distilled water at 37 °C. For this test non-XL scaffolds and cross-linked with different EDC concentration (from 0.1% to 100%) were produced from Sigma and Devro collagens. Samples of approximately 5 mg were accurately weighed (Mb) and submerged in 5 ml of water for different period of time (up to 28 days). After different time intervals samples were removed, dried to constant weight and weighed (Wa). The percentage weight loss was calculated as shown:

Weight loss(%)=100×{(Mb-Ma)/Mb}

Tests were repeated with four parallel samples each.

### 2.8 Mechanical testing

Compressive stress–stain analysis of the scaffolds was performed in the wet stage using a mechanical Hounsfield tester, equipped with a 5 N load cell. Cylindrical specimens, of 8 mm diameter and thickness of 6–8 mm, were cut from the scaffolds and hydrated in deionised water at room temperature for 1 h prior to testing. All compression tests were performed perpendicular to the plane of the scaffold disc at a crosshead speed of 5 mm min^−1^. The linear elastic (Young’s) modulus was obtained via linear regression of the initial linear region of the stress–strain curve. Five replica tests were made for each scaffold.

### 2.9 Cell studies

#### 2.9.1 Film preparation

For cell studies films of ∼8 μm of thickness were prepared by drying 0.5% (w/v) suspensions of protein (Col(S), Col(S)-Gel and Gel) directly in Immulon-2HB 96-well plates (100 μl of slurry/well) for 48 h in a laminar flow cabinet. These were EDC/NHS crosslinked relative to the molarity of collagen carboxylic acid groups as for 3D scaffolds.

#### 2.9.2 Platelet adhesion analysis

Platelets were obtained from human platelet rich plasma provided by the National Health Service Blood and Transplants (NHSBT) authority in accordance with the Declaration of Helsinki. Platelets were prepared by centrifugation for 15 min, 240 g, the pellet discarded and 1 μl of Prostaglandin *E*_1_⋅(100 μg/ml in ethanol) added per ml of platelet supernatant. The platelet suspension was centrifuged at 640 g for 10 min and the cell pellet resuspended in tyrodes buffer (140 mM NaCl, 5.6 mM Glucose, 2 mM MgCl_2_, 0.4 mM NaH_2_PO_4_, 12 mM NaHCO_3_, 2.7 mM KCl, 10 mM HEPES pH 7.4) to a density of 1 × 10^8^/ml. Before platelet incubation, non-specific adsorption to the film or well was blocked with 200 μl of bovine serum albumin (BSA, 5% (w/v) in PBS) for 60 min, and then washed three times with 200 μl of PBS. 100 μl of platelet suspension containing either 5 mM Mg^2+^ or 5 mM EDTA, were added to wells and allowed to attach at room temperature for 30 min. The wells were washed with Tyrodes (200 μl × 3) and then 150 μl of lysis buffer containing PNP phosphatase substrate (81 mM TriSodium Citrate, 31 mM Citric Acid, 0.1% v/v Triton X-00, 1.85 mg/ml PNP substrate, pH 5.4) was added for 90 min at room temperature. After 100 μl of 2 M NaOH was added and the absorbance read at 405 nm (A_405_) using a Fluostar Optima plate reader (BMG Labtech). Integrin mediated platelet attachment was derived by subtracting platelet derived absorbance in the presence of EDTA from the platelet absorbance in the presence of Mg^2+^. Platelet adhesion assays were performed in triplicate and values are reported as means ± standard deviation.

#### 2.9.3 HT1080 adhesion analysis

HT1080 cells derived from a human fibrosarcoma were obtained from the European Collection of Animal Cell Cultures, Porton Down, UK. These were maintained in a humidified incubator with 5% CO_2_ at 37 °C in Dulbecco’s modified Eagle’s medium (DMEM, Sigma–Aldrich) containing 10% fetal bovine serum (Sigma–Aldrich) and 1% streptavidin/penicillin (Life Technologies). Prior to cell adhesion experiments, HT1080s were detached from the cell culture flasks with 0.05% trypsin/0.02% EDTA (GE Healthcare), washed and re-suspended in serum free DMEM.

Collagen films were BSA blocked as for platelet adhesion analysis then 100 μl of HT1080 cells were added at a density of 5 × 10^5^ cells/ml in serum free DMEM containing either 5 mM MgCl_2_ or 5 mM EDTA. After incubation at 37 °C/5%CO_2_ for 40 min loosely bound cells were removed with 3 × 200 μl PBS washes. Bound cells were detected using the phosphatase substrate as for platelet adhesion. Integrin mediated HT1080 attachment was derived by subtracting attachment in the presence of EDTA from attachment in the presence of Mg^2+^. Values indicate means of quadruplicate measurements ± standard deviation.

## 3 Results

### 3.1 Characterisation of collagen slurries

To assess the structural properties of collagen fibre architecture, two different tests were carried out: measurement of the apparent viscosity of 1% (w/v) slurries, which should provide information about molecular weight, and optical observation of collagen suspensions after being treated with fluorescently labelled GFOGER triple-helical peptide, using a confocal microscope.

#### 3.1.1 Rheology

In order to gain insight into the macromolecular structure of collagen from different suppliers (Devro and Sigma), the rheological behaviour of 1% (w/v) suspensions in 0.05 M acetic acid was studied. Col–Gel suspensions prepared from both Col samples and pure Gel solution at 1% (w/v) were also tested under the same conditions.

**Fig. 1:**
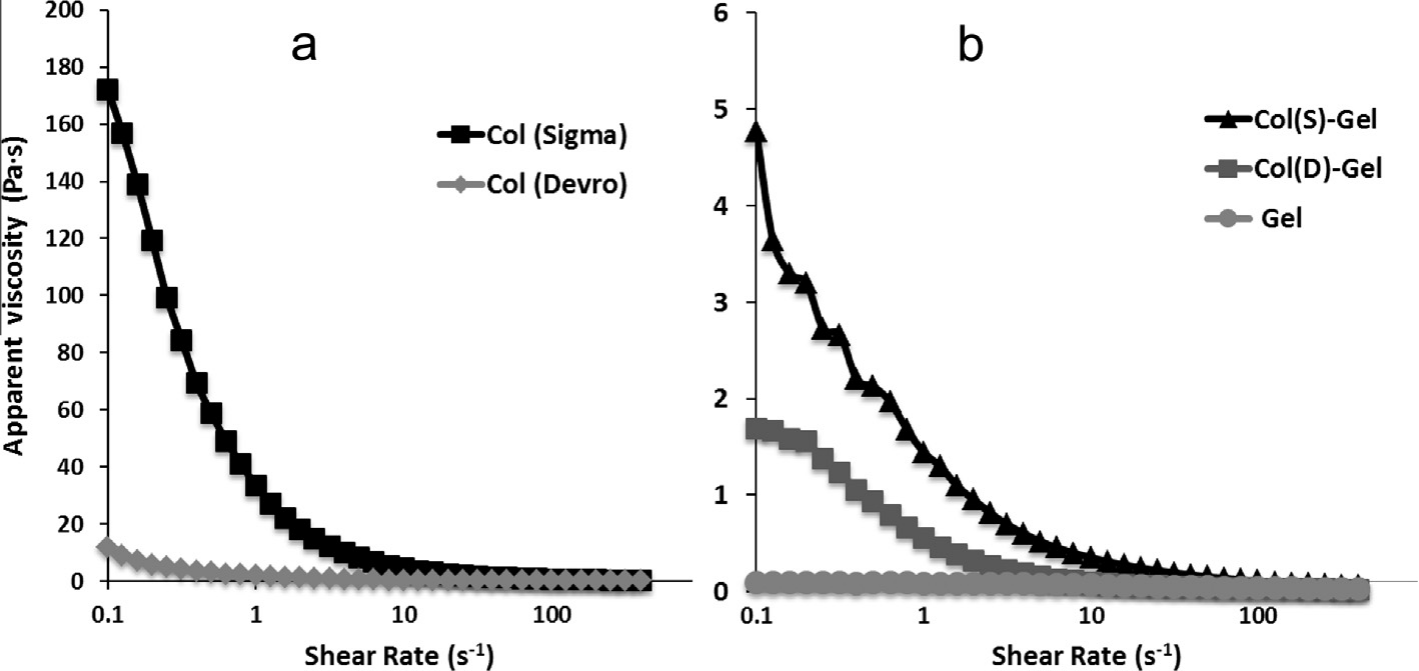
Dependence of the apparent viscosity (Pa s) of Col 1% (w/v) suspensions on shear rate (s^−1^) for Sigma and Devro Type-I collagen at 20 °C.

Fig. 1a shows the apparent viscosity of both collagen suspensions as a function of the applied shear rate. It can be observed that the value of the initial apparent viscosity of Sigma Col is over ten times larger than that of Devro (180 and 12 Pa s, respectively) suggesting that Sigma Col has a higher molecular weight. The level of chain entanglement of the Sigma sample should also be much higher leading to higher values of viscosity. With increase in shear rate, both suspensions experience shear thinning, more evident in the case of the Sigma sample. On increasing the shear rate, the chains become disentangled, aligned and start to slide over each other in non-Newtonian behaviour. At shear rates over 10 s^−1^ both Col samples became fluid with very low viscosity values. The introduction of Gel (50% w/w) to both types of Col (S and D) results in a significant fall in the initial apparent viscosity of both mixed compositions, this being more notable in Col(S)-Gel: from 180 to 6 Pa s for Col (S) and Col (S)-Gel, respectively (Fig. 1b). Gelatin is denatured collagen, with less organised structure and a shorter chain length, which explains the very low values of the apparent viscosity of its solutions. Incorporation of Gel into mixed compositions should reduce the average molecular weight and therefore the number of entanglements in the resultant suspensions, so decreasing the energy required to orientate and align mixed Col–Gel chains. The decrease in viscosity of the Sigma Col containing composition is so big that the differences in fluidity between mixed samples (Col(S)-Gel and Col(D)-Gel become less evident: 6 and 2 Pa s, for Sigma and Devro containing compositions, respectively.

#### 3.1.2 Confocal studies of peptide attachment to collagen fibres

**Fig. 2:**
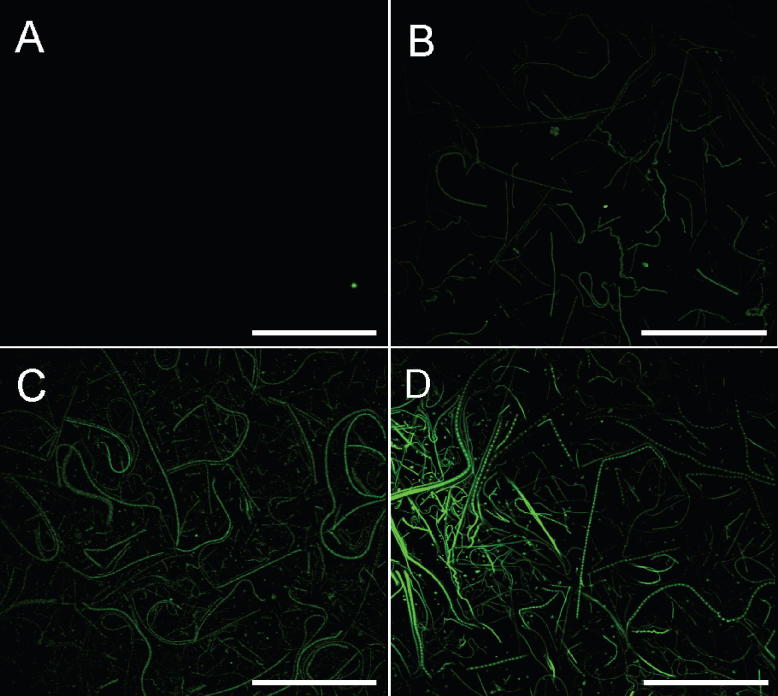
Visualisation of internal structure of collagen suspensions after attachment of GFOGER-FL. Confocal images of different Col samples: (A) monomeric (negative control); (B) Devro; (C) Sigma and (D) Ethicon (positive control). The scale bars are 100 μm.

It has been showed that fluorescein-labelled collagen-mimetic peptides (GPO polymers) can bind to the fibrils in polymeric fibrous collagen [43,44]. Some of us have shown that a fluorescent version of the integrin-binding peptide, GPC[GPP]_5_GFOGER[GPP]_5_GPC, binds similarly to Ethicon collagen fibres [40] thus allowing these fibres to be visualised using a confocal microscope. Observation of how quickly the fluorescence fades gives additional indirect information about fibre length/diameter. Confocal images of collagen slurries, after attachment of the fluorescently marked peptide, are displayed in Fig. 2. Results show that the monomeric collagen (Fig. 2A), as expected, does not bind GFOGER-FL, confirming that the fibril structure is vital for attachment of this triple-helical peptide to collagen [44]. Confocal observation of the macromolecular structure of Col (D) revealed that the fluorescence, due to peptide attachment, faded almost immediately so that it was difficult to achieve good visualisation of its fibre architecture (Fig. 2B). In contrast, fibres of Sigma and Ethicon collagens (Fig. 2C and D) could be clearly observed for a long period of time, indicating greater and stronger attachment of GFOGER-FL. This may be due to the larger fibre diameter, according to the hypothesis that with an increase of diameter/length there are more regions in the collagen chain for peptide attachment [40]. Consistent with this hypothesis, Ethicon showed the highest and most stable level of peptide binding (Fig. 2D), as would be expected, therefore.

Another factor which might influence GFOGER-FL adsorption may be related to the purity of Col samples. Other molecules that remain after Col extraction may coat the fibre surface and hinder the attachment of peptide. The Ethicon sample was produced commercially for wound dressing applications and may be more pure than other collagens, which may also contribute to greater free surface for GFOGER-FL binding and, therefore, visualisation.

### 3.2 Morphology and dimensional properties of scaffolds

**Fig. 3:**
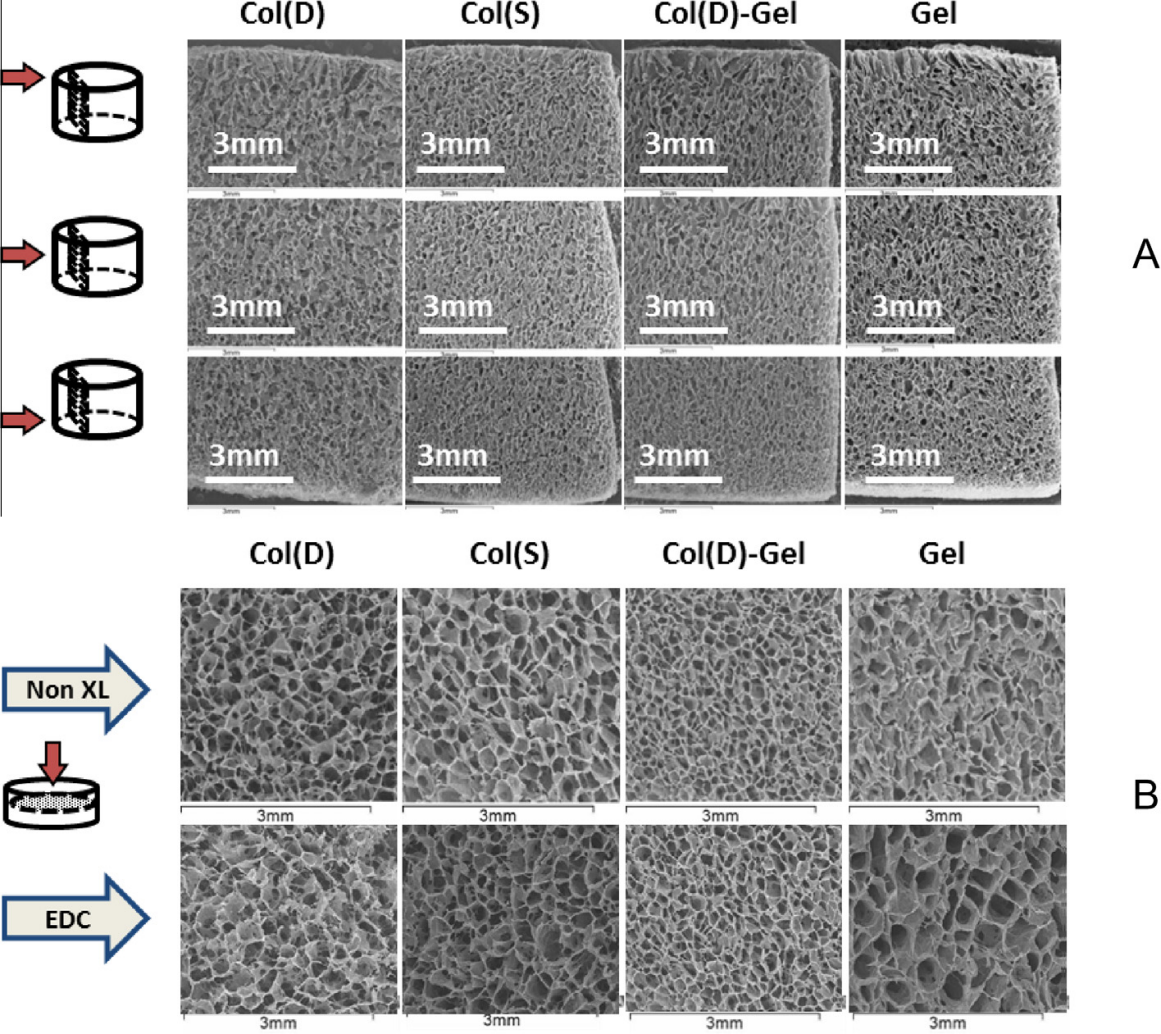
SEM images of scaffolds of different compositions and XL status: (A) non XL scaffolds (different longitudinal cross-sections), (B) influence of cross-linking and Col type (transversal cross-sections).

SEM images of non-XL samples displayed in Fig. 3 show highly porous scaffolds with homogeneous isotropic inner architecture in both longitudinal (Fig. 3A) and transversal (Fig. 3B) cross sections for all scaffolds compositions. Only some pore enlargements on the top and a narrow zone of pore densification on the bottom of scaffold sheet could be observed. The pore dimensions estimated from SEM microphotographs were mostly in the range of 130–260 μm for all studied compositions and XL status. The variation in composition, Col type (S vs D) and XL status do not introduce any appreciable changes in scaffold pore size (Fig. 3B).

Analysis of the data displayed in Table 1 showed that no significant differences in values of dimensional characteristics were detected for non-XL scaffolds of all compositions obtained from both S and D Col. This result is to be expected since these parameters are mostly a function of the slurry concentration from which the corresponding matrices were produced, and in all cases identical 1% (w/v) suspensions were employed to obtain the scaffolds. It is interesting to note that the significant differences in viscosity between S and D collagens slurries had little effect on pore structure. Porosity of ∼99% was obtained for all compositions. These values fall in a desirable range (generally ⩾ 90%) required for 3D cell supports designed for TE applications.

Dimensional parameters were also determined for EDC treated samples of all compositions. Very similar values (99.1–99.2%) were observed for both S and D Col-based scaffolds, mixed compositions and pure Gel matrices after EDC cross-linking with standard conditions (100%) indicating that EDC treatment does not affect porosity values of 3D sponges. These results are in agreement with SEM studies showed that composition and cross-linking have little effect on the scaffold morphology.

### 3.3 Amine group content and degree of cross-linking

**Fig. 4:**
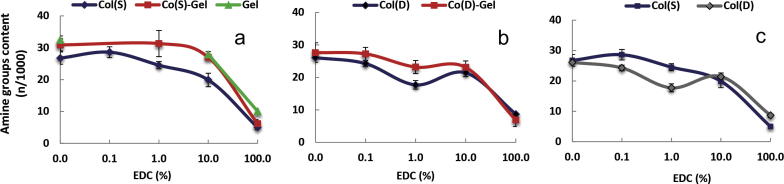
Influence of EDC concentration on the free amine group content on lysine residues of scaffolds of different compositions: (a) Sigma Col-based and Gel samples; (b) Devro Col-based and Gel samples; (c) Col(S) vs Col(D) scaffolds. Results are mean values of three parallel measurements. Error bars represent the standard error.

Profiles of the amount of free amine groups on lysine residues of Col(S and D), mixed Col–Gel and Gel scaffolds before and after carbodiimide treatment with different EDC concentrations are displayed in Fig. 4. Non-XL Gel has a slightly higher number of free amine groups compared to both Col (S and D) samples (33 and 26–27, respectively) and the increase in EDC concentration similarly decreases the amount of free amine group on scaffolds of all compositions and Col types.

After crosslinking with 100% EDC, the free amino group content of all samples decreased significantly, suggesting that the crosslinking procedure was successful. The value of free amine groups in all samples after crosslinking was 5–10. The principal drop in this parameter occurred at EDC concentrations higher than 10%. Table 2 displays values of crosslinking degrees calculated from the free amino group content after each treatment. These results indicated that the percentage of crosslinking of all scaffolds increased almost 4-fold with the increase of EDC concentration from 10% to 100%. Similarities in the profiles of amino group content vs EDC concentration (Fig. 4a–c) suggest that neither the type of Col (S vs D) nor the presence of Gel in scaffold composition significantly affect the extent of scaffold cross-linking reactions.

### 3.4 Influence of EDC concentration and Col nature on scaffold dissolution in water

The objective of this testing was to assess the possible effect of the EDC concentration and the nature of employed Col on the scaffold dissolution in aqueous environment. All scaffold compositions (Col, Col–Gel and Gel) produced from both Col(S) and Col(D) samples were tested for stability in water. These extensive screening studies were carried out in distilled water (instead of using phosphate buffered saline or cell culture medium) for two main reasons. Firstly, the effect of biological media and the presence of enzymes (collagenase) on scaffold degradation behaviour for up to prolonged incubation times has already been assessed in our previous work (unpublished results) and secondly, using distilled water as dissolution medium we avoid an additional sample handling (washing up to 3 times in distilled water to ensure salt removal before drying and weighing) which could alter scaffold integrity, especially for weakly XL samples and after prolonged time of incubation.

**Fig. 5:**
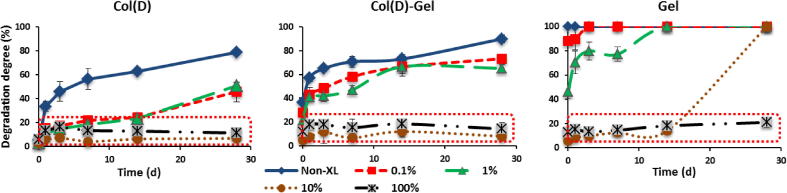
Influence of EDC concentration and composition on dissolution behaviour of Col(D)-based and Gel scaffolds. Results are mean values of four parallel measurements. Error bars represent the standard error.

Both Col(S and D), mixed and pure Gel samples were tested for their resistance to dissolution after EDC treatment with different concentration. As an example of the effect of EDC crosslinking concentration on scaffold stability in water, the dissolution profiles for Col(D)-type scaffolds, mixed compositions and Gel samples are shown in Fig. 5. It could be observed that Col(D) and mixed scaffolds crosslinked with full (100%) and 10% EDC concentrations possess high resistance to dissolution over all studied period (up to 28d). Gel samples treated with the same crosslinking conditions were also stable in water but for up to 14d. DD were very similar and time independent for all compositions over a period of 14d which is a sign of the high crosslinking density achieved by chemical treatment with these conditions. Very low EDC concentrations (0.1% and 1%) still could effectively stabilise Col(D) scaffolds (up to 14d) and at less extent mixed Col–Gel (up to 7d). Gel showed some improvement in resistance to dissolution at 1% EDC after 1 h in water (DD ∼ 40% comparing to complete dissolution of non XL sample) but at 24 h mass loss was already very high (>65%).

**Fig. 6:**
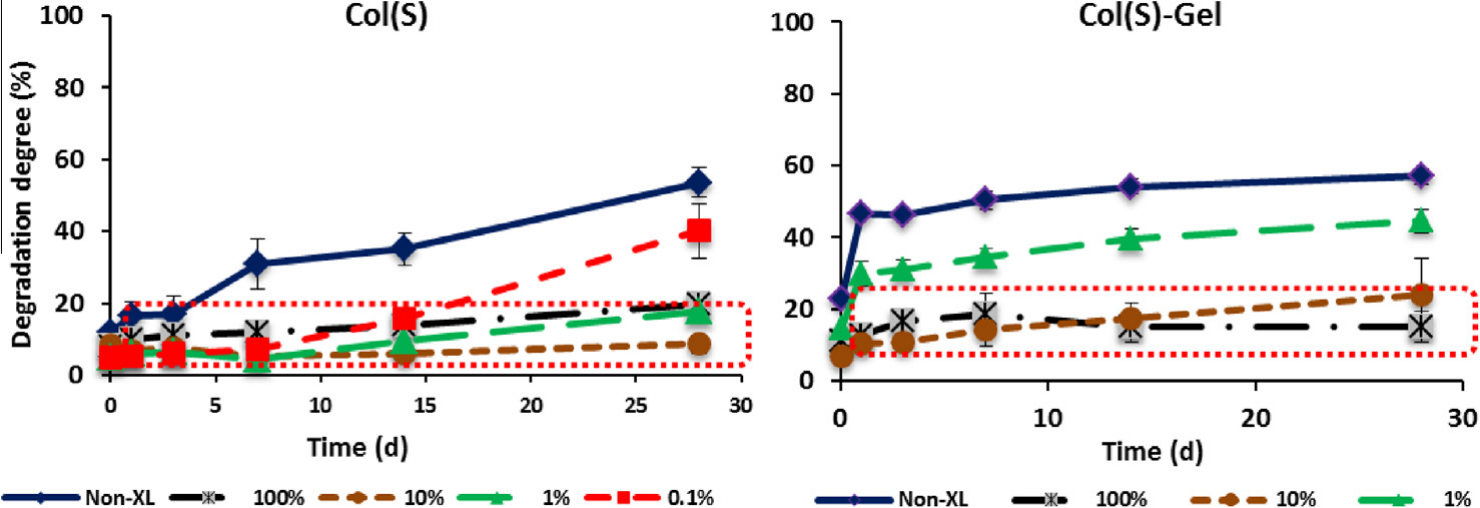
Dissolution profiles of Col(S)-based scaffolds cross-linked with different EDC concentration. Results are mean values of four parallel measurements. Error bars represent the standard error.

**Fig. 7:**
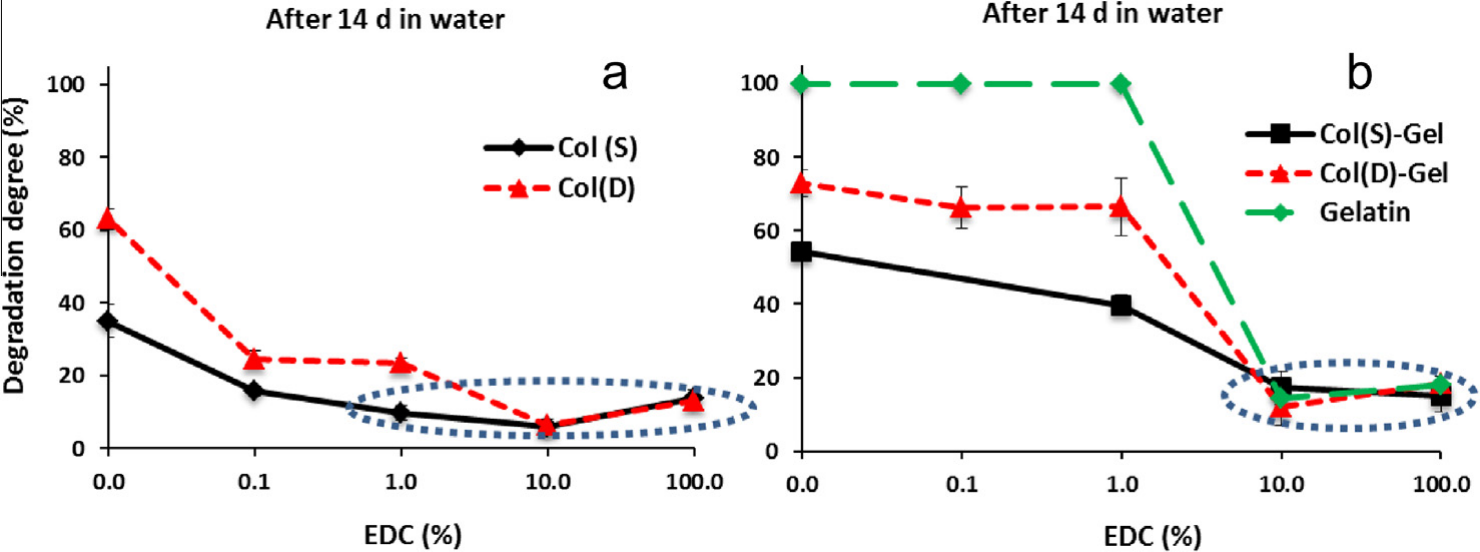
Mass loss: (a) Col(S vs D) and (b) mixed Col(S vs D)-Gel and Gel scaffolds after 14d in water. Results are mean values of four parallel measurements. Error bars represent the standard error.

The influence of Col nature on dissolution stability of EDC treated scaffolds could be observed in Figs. 6 and 7. Comparing results in Fig. 6 with those in Fig. 5 it can be concluded that dissolution profiles and DD values are very similar for both Col types (S and D) over all incubation period (up to 28d) when these scaffolds are treated with 10% and 100% EDC concentrations.

At lower EDC concentrations (0.1% and 1%) mass loss is similar up to 7d and then Col(D) matrices gradually decrease their resistance to dissolution while Col(S) samples are stable for up to 28d when cross-linked with 1% EDC. Non-XL Col(S)-Gel scaffolds were also more resistant to dissolution that mixed Col(D)-based samples (∼60% and ∼80% of dissolution after 28d for Col(S) and Col(D)-based matrices, respectively). The highlighted areas show how similar degradation behaviour may be seen when the EDC concentration is lowered from the standard 100% level. Fig. 7a and b confirm that EDC at concentration of 10% onward can provide the same stability to Col scaffolds independently on Col origin, to the mixed Col–Gel composition and to pure Gel scaffold and that the differences in resistance to dissolution only became evident with the decrease of EDC concentration, for both pure Col and mixed compositions. At 14d all scaffolds show the same DD degrees for EDC concentration of ⩾10%. The decrease in EDC content from 10% to 1% significantly destabilised mixed compositions (more noticeable in Col(D)-Gel samples) while pure Gel matrices crosslinked with 1% EDC were completely solubilised at this time point.

### 3.5 Mechanical testing of different scaffold compositions

Compressive properties are of greater interest when studying the impact of scaffold mechanics on cellular activity, because cells, through their action, tend to bend and buckle individual struts within the scaffold [32,45,46]. The linear elastic (Young’s) modulus (*E*^∗^) calculated from stress–strain analysis gives precisely the measure of the resistance of the struts to bending and buckling under compression.

**Fig. 8:**
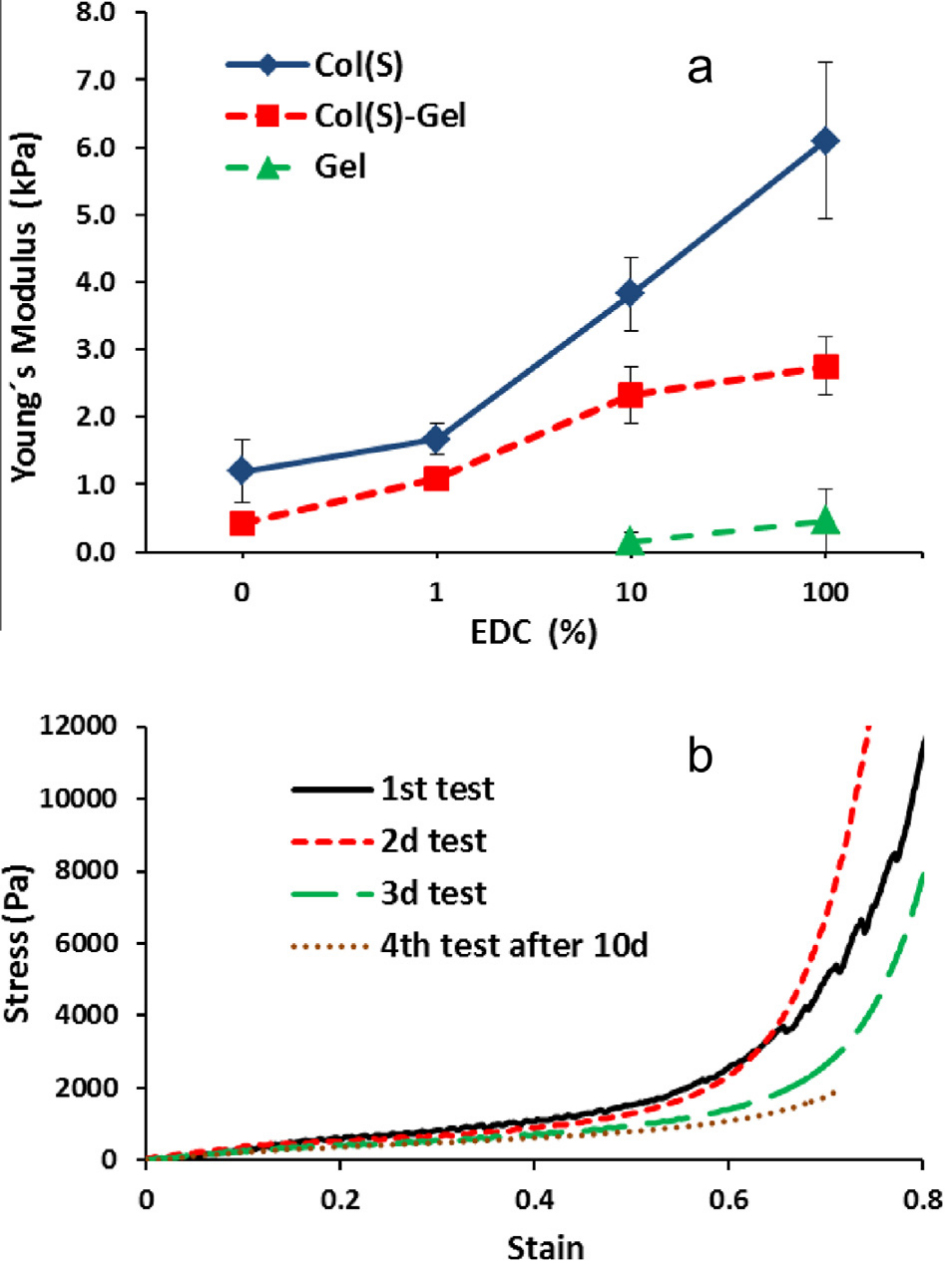
Influence of EDC concentration on the compressive modulus (*E*^∗^) of the scaffolds of different compositions (a) and typical profiles of compressive stress–strain curves observed for hydrated Col(S) scaffolds after repeated compressions of the same specimen over the entire strain range (e: 0–0.8) (b). Results are mean values of five parallel measurements. Error bars represent the standard error.

Mechanical testing was carried out on all compositions (Col, Col–Gel and Gel) treated with different EDC concentrations. Col(S) was selected to assess the influence of different cross-linking treatments on scaffold mechanics. The values of *E*^∗^ for Sigma Col-containing samples and pure Gel showed that EDC cross-linking at different concentrations increases this parameter for all studied scaffolds but to a different extent (Fig. 8). Gel samples treated with 1% EDC were structurally too weak to be reliably assessed in compression. All EDC-modified at standard cross-linking condition (11.5 mg/ml EDC, 100%) matrices displayed the highest Young’s Modulus and 10-fold reduction in EDC/NHS concentrations still provides good mechanical stability for Col-containing scaffolds compositions. The introduction of Gel drastically decreases mechanical strength of scaffolds even after crosslinking with maximum EDC concentration.

To assess the scaffold’s behaviour under successive compressions the same sample specimens were repeatedly compressed at least three times and, in a case of Col(S), also after 10 days in water. Example of stress–strain curves (Fig. 8b) corresponding to this testing showed no significant changes in their profiles up to a strain of 0.4 (curves almost overlay each other). Young’s moduli calculated from the curves after successive compressions reveal, however, some differences in scaffold elastic properties, more evident for weakly EDC XL scaffolds. For example, Col scaffolds modified with higher EDC concentration (10% or 100%) were very stable mechanically even after 4 repeated compressions (3 consequent followed by one after 10d of incubation in water). No visual changes were observed in Col pore structures though their *E*^∗^ were reduced in ∼20% and ∼30% for 10% and 100% EDC concentrations, respectively. Elastic moduli of Col–Gel samples decreased in ∼30–40% after third successive compression. The value of *E*^∗^ for Col 1% EDC XL samples experimented significant decrease in ∼65% after 10d of incubation in water while 3D structures of non-XL Col and Col–Gel or 1% EDC XL mixed Col–Gel scaffolds were too weak to be accurately tested.

### 3.6 Cellular interactions

**Fig. 9:**
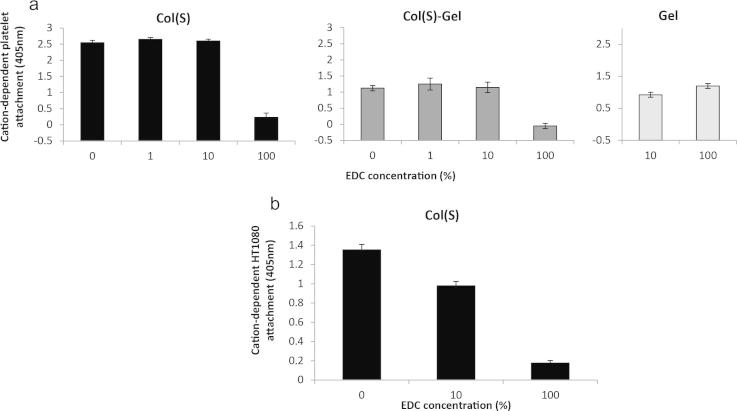
Integrin mediated platelet (a) and HT1080 cell (b) attachment to films prepared from sigma collagen (Col(S) – black bars), 50:50 sigma collagen:gelatin (Col(S):Gel – dark grey bars) and gelatin (Gel – light grey bars) treated with increasing concentrations of EDC where 100% equates to 2:5:1 M ratio of EDC:NHS:collagen COOH groups. Results are mean values of quadruplicate measurements. Error bars represent standard deviation from the mean.

Platelets can adhere to collagen selectively through integrin α_2_β_1_ but also a non-integrin receptor, GPIV. Therefore platelet binding is an ideal experimental system to measure the binding of an individual integrin class, namely integrin α_2_β_1_, in isolation. Integrin α_2_β_1_ binding to collagen occurs through the high affinity, structurally sensitive GFOGER motif on triple helical collagen. Therefore in order to determine if EDC crosslinking of the carboxylic acid group on the *E* of GFOG*E*R can inhibit integrin engagement with this motif we measured integrin-mediated platelet binding to collagen sample. This was achieved by subtracting the binding observed in the presence of the cation chelator EDTA (non-integrin-mediated binding) from the binding observed in the presence of the integrin stimulating cation Mg^2+^ (integrin-mediated biding) (Fig. 9a). For this analysis thin films were prepared to mimic the 3D struts, mitigating complicating cellular effects from a 3D system, allowing direct measurement of the cell responses to chemically modified collagen. On Col(S) films the degree of integrin-mediated platelet adhesion was reduced by conventional (100%) EDC crosslinking conditions compared to a non-crosslinked control (Abs_405_ of 2.55 ± 0.07 and 0.23 ± 0.13 for non-Xl and 100% EDC respectively). By contrast 1% or 10% of conventional EDC crosslinking did not significantly affect integrin-mediated platelet engagement with collagen films, yielding absorbances of 2.65 ± 0.06 and 2.60 ± 0.05 respectively. Gelatin incorporation reduced integrin α_2_β_1_-mediated platelet interactions with the resulting Col(S)-Gel samples compared to Col(S) (Abs_405_ of 1.12 ± 0.08). Crosslinking with 1% or 10% EDC/NHS did not alter the degree of integrin-mediated platelet adhesion whereas crosslinking with 100% EDC/NHS significantly inhibited integrin-mediated platelet adhesion to Col(S)–Gel films giving an absorbance of −0.048 ± 0.08. It should be noted that this negative reading is a result of a slightly higher absorbance for the EDTA treated platelets than the Mg^2+^ treated platelets. The extreme solubility of Gel restricted platelet binding analysis to 10% and 100% EDC crosslinked samples. These bound to approximately the same number of platelets as the mixed composition Col(S)–Gel which was not sensitive to 10% or 100% EDC crosslinking.

HT1080 cells utilise integrin α_2_β_1_ exclusively for binding to GFOGER making them an ideal system to analyse cell-associated integrin _21_ interactions with collagen films. As gelatin incorporation reduced the level of integrin mediated platelet adhesion the Col(S)-Gel and Gel samples were not further analysed. Instead Col(S) alone was used to determine the influence of EDC crosslinking on HT1080 fibrosarcoma cell adhesion. HT1080 cell adhesion to Col(S) was inhibited from 1.35 ± 0.06 on non-crosslinked Col(S) to 0.18 on 100% EDC/NHS crosslinked (Fig. 9b). By contrast 10% EDC/NHS crosslinking only moderately affected integrin mediated HT1080 cell attachment giving an absorbance of 0.98 ± 0.04.

Together these date indicate that by utilising lower EDC/NHS crosslinking conditions it is possible to preserve native-like integrin _21_ mediated cell interactions with collagen-based materials.

## 4 Discussion

### 4.1 Characteristics of collagen suspensions

Rheological and peptide binding studies indicate that significant differences exist in the fibre length and diameter of collagen depending upon its origin. The Sigma sample possesses a higher molecular weight with greater fibre diameter than that of Devro. Although both collagen samples are of bovine type, Sigma was extracted from bovine Achilles tendon, a high load tissue, particularly resistant to tension, while Devro was derived from dermal tissue, which is soft, flexible and only sustains lower loads. Tendon collagen can be extracted in quite good purity simply by dissection, whereas skin collagens require more complex processing, and tendon fibres are known to be quite long and of large diameter. These changes in tissue properties, as well as in extraction/purification methods, may explain the differences found in the macromolecular parameters and organisation of collagen chains of Sigma and Devro samples. This, in turn, may explain the differences in the material properties of scaffolds produced from these collagen samples, such as their degradation degree and resistance to compression.

### 4.2 Morphology

It is well known that polymer scaffolds for use in cell culture must be highly porous with large surface/volume ratios to provide sufficient space for cell growth and proliferation [32]. The pore size and interconnectivity are important in allowing cell infiltration, vascularisation and the diffusion of nutrients and oxygen, and the scaffold porosity should be high and interconnected to prevent obstruction of the material, which may lead to necrosis *in vivo* [47,48]. Scaffold production conditions chosen in this work allowed highly porous (porosity ∼99%) 3D scaffolds with interconnected inner architecture to be obtained for all compositions studied. The pore diameters, similar for all scaffolds, were mostly between 130 and 260 μm. Crosslinking with EDC has not significantly altered the scaffold inner architecture in such a way as to produce a detrimental effect on pore size and porosity. The pore size was within a range suitable for the growth of myocytes, endothelial cells and fibroblasts as reported in previous research [48–50]. This means that the morphology of all protein matrices was appropriate for their use in myocardial TE applications.

### 4.3 Amine group analysis and cell interactions

Estimation of the free amino group content of all scaffold compositions under different XL conditions allowed the extension of EDC-mediated bonding in macromolecular chains of proteins to be monitored and controlled. It was shown that, by varying EDC content, scaffolds with a variety of crosslinking degrees could be obtained, and that this process was almost independent of Col precedence (S or D) and Gel addition. The consumption of free amine groups during EDC-treatment should result in proportional reduction of free carboxylate anions on glutamate or aspartate residues (according to XL mechanism). This in turn might lead to the reduction of the availability of cell binding sites on scaffold proteins with the consequent impact on the biological properties of the resultant samples. Notably, EDC treatment could modify the E on the cell binding GFOGER motif in collagen that bind specifically to the four collagen-binding integrins. Furthermore, EDC could modify the RGD motifs that are present, but sterically hindered from integrin binding, in the latent form in collagen but become active on denaturation (in gelatin) and become competent to bind some of the fibronectin-binding integrins [51].

The possibility of EDC chemical modification of integrin binding motifs on collagen-based samples was tested by platelet and HT1080 fibrosarcoma binding assays. Both cell types bind to collagen via integrin _21_ which is sensitive to the presence of the GFOGER motif. These showed that integrin _21_ dependent cell attachment to collagen was ablated by 100% EDC conditions, supporting the conclusion that EDC treatment chemically modifies integrin-binding sites on the collagen-based materials. To compensate a 10-fold lower EDC crosslinking regime can be utilised, allowing full platelet and increased HT1080 integrin-mediated interactions. Addition of gelatin to the collagen composition reduced the degree of integrin mediated platelet binding. This is presumably due to the effective dilution of triple helical GFOGER motifs on the native collagen with the addition of RGD motifs in gelatin. As for pure Col(S) compositions, 100% EDC crosslinking ablated platelet attachment but lower EDC conditions (⩽10%) allowed comparable platelet attachment to the non-XL controls. Therefore the cell attachment data are in agreement with the amine content analysis showing that consuming amine groups, and by extension carboxylic acid groups such as on the *E* of GFOG*E*R, impacts on the bioactivity of the resultant materials. This data provides a maximal degree of side chain consumption that is compatible with bioactivity. In the case of Col(S) compositions this equates to ∼26% of amine crosslinking using a molar ratio of 0.5 EDC:0.2 NHS:1 COO^−^(Col) to retain cellular biorecognition.

### 4.4 Dissolution in water

Results of dissolution studies in water showed that EDC treatment at concentration of 10% onwards effectively stabilised all studied compositions, almost independently of Gel presence or Col origin which is in agreement with the results of degrees of cross-linking calculated from the free amino group content. The nature of the Col employed plays a significant role in scaffold stability only for untreated matrices or for those cross-linked with very low EDC concentration (1% or less). The presence of Gel requires a higher concentration of carbodiimide to provide stability of mixed or pure Gel compositions to prevent loss of scaffold material to the media at prolonged incubation times in an aqueous environment. Scaffolds obtained from Sigma Col are relatively stable in a wet environment even without additional chemical treatment and may be employed without EDC cross-linking for their use as cell supports for up to 7 days. The differences in dissolution properties between D and S collagens are more evident at lower EDC. By changing EDC concentrations further control over the degradation kinetics may be achieved. Variation of the Col origin opens an additional way of tuning/tailoring the final properties of Col-based scaffolds towards specific TE applications.

### 4.5 Compression modulus

It has already been demonstrated that scaffold microstructure and stiffness affects the bioactivity of these systems [52]. Previous studies into the mechanical stiffness and strength have shown that these properties were increased after scaffold crosslinking [1,2,6,10,37,46]. Tests, conducted in this study on the effect of the EDC XL conditions on the mechanical performance of scaffolds revealed that both crosslinking density and composition have significant impact on the compressive modulus (*E*^∗^) of the resultant 3D matrices. EDC treated Col scaffolds exhibited higher values of these parameters compared to Gel-containing samples and the increase in EDC concentration enhanced the values of *E*^∗^. It is known that the Young’s modulus of 3D scaffolds depends upon the relative density of the sponge, the elastic modulus of struts (solid from which scaffolds are made) and a constant related to the pore geometry. As relative density and cell geometry are the same for all samples (no changes in morphology and dimensional properties were found after EDC treatment for all compositions) it is logical to assume that the increase in strut stiffness is a results of variations in composition (from Gel to Col) and in the enhancement of the degree of XL due to bond formation within scaffold struts induced by EDC.

## 5 Conclusions

This research addresses the question of how the composition and crosslinking of collagenous biomaterials affect their physical properties and the number of cell binding sites on glutamate or aspartate residues (proportional to free amine groups on lysine residues), crucial for integrin mediated cell adhesion to scaffold surfaces. Highly porous (porosity ∼99%) Col (from different suppliers), Gel and mixed Col–Gel (50–50% wt.) 3D matrices with homogeneous inner architecture and average pore diameter (130–260 μm), suitable for soft tissue TE, were produced using a freeze drying technique. The effect of Col origin and of the variation of EDC crosslinking conditions (down to very low EDC content) on the morphology and the relevant material properties of scaffolds of different composition was established. Characterisation of Col slurries, obtained from Col from different suppliers (Sigma vs Devro), in terms of rheological properties and peptide attachment to Col fibres, allowed insight into their macromolecular structure and provided an explanation for the results of material testing. It was shown that scaffold mechanics was highly dependent upon the EDC content for all compositions, and that in order to achieve stable resistance under successive compressions the 3D matrices should be treated with 10% EDC or more, especially in the case of Gel-derived samples. It was also found that irrespective of the scaffold constituents used, it was possible to reduce the amount of EDC by at least 10 times without loss of scaffold stability in water. Consistent with the lower chemical modification of vital carboxylic acid groups, this 10-fold reduction in EDC crosslinking permitted integrin mediated cell interactions with the scaffold. It may be concluded, therefore, that variation of EDC concentration provides a simple method of modulation of the physical and biological properties of scaffold material. Further tuning of scaffold chemistry, mechanics and biological characteristics may be achieved by changing Col origin and/or introducing Gel to the scaffold composition.

## Figures and Tables

**Table 1:** Average values of density and porosity (pore volume fraction) of scaffolds obtained from 1% (w/v) suspensions.

Composition	Density (g/cm^3^)	Relative density	Pore volume fraction (%)
Col(D)	0.0110 ± 0.0003	0.0084 ± 0.0002	99.16 ± 0.02
Col(D)–Gel	0.0110 ± 0.0004	0.0085 ± 0.0003	99.15 ± 0.03
Col(S)	0.0108 ± 0.0001	0.0083 ± 0.0001	99.17 ± 0.01
Col(S)–Gel	0.0108 ± 0.0004	0.0083 ± 0.0003	99.17 ± 0.03
Gelatin	0.0112 ± 0.0006	0.0086 ± 0.0005	99.14 ± 0.05

Results are expressed as mean values of five parallel measurements ± standard errors.

**Table 2:** Degree of crosslinking of scaffolds calculated from free amino group content after treatments with different EDC concentration.

EDC concentration	Degree of crosslinking (%)				
(%)	Col(S)	Col(D)	Col(S)–Gel	Col(D)–Gel	Gel
100	81 ± 3	67 ± 5	80 ± 3	75 ± 3	69 ± 3
10	26 ± 10	18 ± 8	13 ± 4	16 ± 6	14 ± 4
1	8 ± 3	32 ± 9	2 ± 10	16 ± 5	
0.1	0 ± 10	7 ± 6		1 ± 6	

Results are expressed as mean values of three parallel measurements ± standard errors.
